# Biotechnology Production of Cell Biomass from the Endangered *Kickxia elatine* (L.) Dumort: Its Untargeted Metabolomic Analysis and Cytotoxic Potential Against Melanoma Cells

**DOI:** 10.3390/biomedicines13061382

**Published:** 2025-06-04

**Authors:** Anastasia Aliesa Hermosaningtyas, Ewa Totoń, Anna Budzianowska, Natalia Lisiak, Aleksandra Romaniuk-Drapała, Dariusz Kruszka, Monika Rewers, Małgorzata Kikowska

**Affiliations:** 1 Doctoral School, Poznan University of Medical Sciences, 70 Bukowska St., 60-812 Poznan, Poland; 88428@student.ump.edu.pl; 2 Laboratory of Pharmaceutical Biology and Biotechnology, Department and Division of Practical Cosmetology and Skin Diseases Prophylaxis, Poznan University of Medical Sciences, Collegium Pharmaceuticum, 3 Rokietnicka St., 60-806 Poznan, Poland; abudzian@ump.edu.pl; 3 Department of Clinical Chemistry and Molecular Diagnostics, Poznan University of Medical Sciences, Collegium Pharmaceuticum, 3 Rokietnicka St., 60-806 Poznan, Poland; etoton@ump.edu.pl (E.T.); nlisiak@ump.edu.pl (N.L.); aromaniuk@ump.edu.pl (A.R.-D.); 4 Institute of Plant Genetics, Polish Academy of Sciences, 34 Strzeszyńska St., 60-479 Poznan, Poland; dkru@igr.poznan.pl; 5 Department of Biotechnology, Faculty of Agriculture and Biotechnology, Bydgoszcz University of Science and Technology, Kaliskiego Ave. 7, 85-796 Bydgoszcz, Poland; mrewers@pbs.edu.pl

**Keywords:** melanoma cell, cytotoxic activity, callus, cell suspension culture, phytochemical and metabolomic analyses, nuclear DNA content

## Abstract

**Background:** Melanoma is a malignant tumor of melanocytes with an increasing incidence worldwide. Plant-based products are rich in bioactive compounds, offering low toxicity and accessible alternatives for melanoma treatment. A biotechnological approach to obtaining plant-derived produce ensures continuous and high-yield production of medicinally valuable biomass. **Objectives:** This study aimed to induce and optimize the growth of homogenous callus cultures of *Kickxia elatine* (L.) Dumort., consequently established a cell suspension culture with a high biomass growth rate, analyzed the phytochemical compositions, and assessed the cytotoxic activity against melanoma cells. **Methods/Results:** Callus cultures were induced under controlled in vitro conditions on Murashige and Skoog (MS) media supplemented with 2.0 mg L^−1^ Dicamba and 2.0 mg L^−1^ 2,4-Dichlorophenoxyacetic acid. The selected callus lines exhibited a high growth index (351.71% ± 27.77) and showed a homogeneous morphology, beige colour, and had friable and watery characteristics. A combination of auxin and cytokinin was found to enhance biomass production significantly. Phytochemical investigations putatively annotated major compounds, including benzoic acid derivatives, phenolic glycosides, phenylpropanoic acids, hydroxycinnamic acid derivatives, and tyrosol derivatives. Methanolic extract (KE-Ex) and 40% methanolic fraction (KE-40Fr) were prepared and tested for cytotoxicity against human fibroblast (MRC-5) and melanoma (MeWo) cell lines using direct cell counting and MTT assay. The crude extract exhibited the strongest cytotoxicity effect on MeWo cells, with IC_50_ values of 125 ± 8 µg mL^−1^ after 48 h and 117 ± 7 µg mL^−1^ after 72 h of treatment. **Conclusions:** The extract demonstrated a time- and dose-dependent cytotoxic effect, making it a potential candidate for melanoma treatment.

## 1. Introduction

Melanoma is a malignant tumour of melanocytes usually seen in the skin and has the poorest prognosis among other types of skin cancer [1]. In Europe, the occurrences of melanoma cancer reached <25 cases per 100,000 individuals. Furthermore, a study conducted between 2000 and 2020 in Poland discovered that the mortality rate has increased from 3.60 to 4.30 per 100,000 population [1,2]. Therefore, studies on searching for melanoma cancer treatment without side effects that are easily accessible to patients are needed. Plant-based products have been demonstrated to be essential resources against melanoma disorders, are less hazardous to the human body, and enhance patients’ quality of life [3].

*Kickxia elatine* (L.) Dumort (syn. *Antirrhinum elatine* L.) is a perennial herb/weed that belongs to the Plantaginaceae (formerly Scrophulariaceae) family. Recent observations in Switzerland [4], Poland [5], and Germany [6] emphasize that this taxon is becoming rare and endangered. In Potter’s new cyclopedia of botanical drugs and preparations, the species is listed as *Linaria elatine* and is mentioned to be capable of use as an astringent [7]. The infusion of *K. elatine* taken internally was recommended for internal bleeding, profuse menstruation, and nosebleeds. Similarly, within the Balkan region and Western Ghats of India, *K. elatine* has been used in conventional medicine as a sedative to help skin cuts, wound healing, and cure hemorrhages and lacrimation. In eastern Serbia, the whole *K. elatine* plant is used to help children with enuresis nocturna (bedwetting) issues [8,9,10]. Italian ethnobotanical studies noted that the leaf of *K. elatine* is commonly used to treat foot hyperhidrosis [11]. Despite its medicinal potential, only a few studies have investigated the chemical properties of *K. elatine* [10,12,13,14]. Handjieva et al. (1994) reported the presence of particular iridoid glycosides: kickxioside, antirrinoside, linarioside, antittide, mussaenosidic acid, 5-*O*-menthiafoloylkickxioside, and kickxin [14]. Moreover, Yuldashev et al. (1996) isolated demethoxycentaureidin 7-*O*-β-D-glucoside, demethoxycentaureidin 7-*O*-rutinoside, pectolinarin, and acetylpectolinarin from the extract of the epigeal part of *K. elatine* [10]. Nonetheless, the presence of metabolites derived from this species may hold promise for cancer research.

Due to its rare and protected status, biotechnologically produced *K. elatine* biomass provides a sustainable source for phytochemical and biological analysis. Plant cell cultures, for instance, could produce chemical compounds for pharmaceuticals, agricultural, horticultural, and cosmetic purposes [15,16]. Furthermore, cell culture-derived products are not constrained by source accessibility, benefit from a controlled and optimized cultivation process, and are independent of geographic location or climatic changes [17,18]. Developing plant cell cultures utilizing biotechnology offers a promising alternative for ensuring constant material availability and yield, as well as generating homogeneous biomass with consistent phytochemical composition for these species [19,20].

The present study attempts to perform morphological, cytogenetic, and phytochemical estimation of *K. elatine* cell cultures, which, to our knowledge, has yet to be reported. To properly assess the impact of selected parameters on biomass growth, the biotechnological parameters of the callus/suspension were estimated. The obtained cell biomass will be further studied for large-scale production. Thus, the results could be valuable insights into applying cell cultures for high-value compound production. Furthermore, this study investigates the cytotoxic activity of *K. elatine* extract from their cell biomass in the human melanoma cell line (MeWo) and a human lung fibroblast in vitro model (MRC-5).

## 2. Materials and Methods

### 2.1. Plant Material and In Vitro

*Kickxia elatine* (L.) Dumort seeds were obtained from Bremen (Free Hanseatic City of Bremen, Germany), belonging to the Botanical Garden and Rhododendron-Park Bremen (Botanischer Garten und Rhododendronpark). The seed lot number XX-0-BREMR-XXXX/4239 was used for the experiments. For aseptic culture initiation, the seeds were disinfected using 70% ethanol and 50% commercial bleach. The disinfected seeds were transferred to Murashige and Skoog solid medium [21] without plant regulators (PGRs) and placed in the dark for 14 days to induce germination. The seedlings were the source of callus induction. The cultures were placed in a growth room under a 16:8-hour photoperiod at 20 ± 2 °C.

### 2.2. Callus Culture

Callus induction was started using the selected explants isolated from the in vitro-obtained seedlings: petiole, leaf, stalk, and root. Explants were placed on solidified MS media (7.2 g L^−1^ agar) with selected plant growth regulators (PGRs). The media were supplemented with 2,4-dichlorophenoxyacetic acid (2,4-D; Sigma-Aldrich, St Louis, MO, USA), 3,6-dichloro-2-methoxybenzoic acid (Dicamba, Dic; Sigma-Aldrich, St Louis, MO, USA), and 1-phenyl-3-(1,2,3-thiadiazol-5-yl)-urea (thidiazuron, TDZ; Sigma-Aldrich, St Louis, MO, USA) at different concentrations and combinations.

Calluses obtained in the primary cultures, characterised by good growth and morphology, were transferred to fresh media. Subsequent passages of callus cultures took place every 30 days. The initial weight (W0) and final weight (WX) of the callus culture were measured. The data were collected from ten replications. The growth index [GI] was calculated after three passages during three consecutive subcultures from the following formula: GI(%) = ((WX − W0)/W0) × 100. The morphology of the callus was assessed based on colour, shape, homogeneity, and texture. The cells were examined using a Leica DM750 RH microscope and Leica Application Suite version 3.1.0 software (Leica Microsystems Inc., Wetzlar, Germany).

### 2.3. Cell Suspension Culture Initiation and Maintenance

The cell suspension culture of *K. elatine* was initiated by inoculating 1.8 g of stabilized callus into 30 mL of MS liquid media, supplemented with Dic 2.0 mg L^−1^ + 2,4-D 2.0 mg L^−1^ and 3% sucrose. The cells were passaged every 30 days by transferring them into fresh media with a ratio of 3:10. Microscopic observations were conducted to evaluate the type of cell growth and contamination levels. The growth curve of the *K. elatine* cell suspension culture was made by measuring the fresh and dry weight every three days in a 30-day cycle with three biological replications (on a total of 30 Erlenmeyer flasks). Cell suspension cultures of *K. elatine* were maintained on a rotary shaker at 110 RPM in a controlled room with a 16:8-hour photoperiod at 20 ± 2 °C. The fresh biomass was dried at 30 °C for three days and weighed to measure the dry weight.

### 2.4. Flow Cytometry

The nuclear DNA content was estimated using flow cytometric analysis on in vitro-derived shoots passage 5, cell suspension cultures passage 3, and seeds (control). The plant materials were immersed in a nuclei isolation buffer solution (0.1 M Tris, 2.5 mM MgCl_2_·6H_2_O, 85 mM NaCl, 0.1% (*v*/*v*) Triton X-100, pH = 7.0) supplemented with 50 µg mL^−1^ of propidium iodide and 50 µg ml^−1^ ribonuclease A. Next, they were cut to release the nuclei using a sharp razor blade and subsequently filtered through a nylon mesh with a diameter of 50 μm to separate the particles. At least 5000 nuclei were then examined using the CyFlow Ploidy Analyser (Sysmex Partec GmbH, Görlitz, Germany) with linear signal amplification. The histograms were evaluated using the CyView 1.6 program (Sysmex Partec GmbH, Görlitz, Germany). Nuclear DNA content was quantified by utilizing the linear correlation between the ratio of the 2C peak positions of the species being analyzed and the internal standard *Solanum lycopersicum* cv. Stupicke (1.96 pg/2C) [22]. This correlation was visualized on a histogram of fluorescence intensity. Genome size was estimated from three biological replications.

### 2.5. Extraction of Secondary Metabolites for Metabolomic Analysis

Callus and cell suspension biomass (±100 mg, respectively) were pulverised using RETSCH MM 400 Mixer Mill (Retsch GmbH, Haan, Germany). The samples were aliquoted with 100% methanol in Eppendorf tubes, and 100 μg mL^−1^ formononetin (Sigma-Aldrich, St Louis, MO, USA) was added as the internal standard for UPLC-MS. The samples were mixed thoroughly for 20 min and then sonicated for 15 min in an ultrasonic water bath (Bandelin Sonorex, Bandelin Electronic GmbH and Co., Berlin, Germany). Samples were then centrifuged at 12,000 rpm for 10 min. About 800 μL of supernatants were collected and vacuum-dried using Savant SC100 SpeedVac (Savant Instruments, Farmingdale, New York, USA). Formic acid solution of 1 mL of 0.1% *v*/*v* was added to the vacuum-dried samples and then put into an ultrasonic water bath for 10 min at room temperature. Finally, the samples were centrifuged at 15,000 rpm for five minutes. Oasis HLB Cartridges (Waters Corp., Milford, MA, USA) performed methanolic extractions from all supernatants. Three biological replications were used for the extraction from the callus culture.

### 2.6. UPLC-HRMS/MS Analysis and Data Processing

The extracts were analyzed using an ultra-performance liquid chromatography system (UPLC) coupled with high-resolution tandem mass spectrometry (HRMS/MS, Q-Exactive Orbitrap, Thermo-Fisher Scientific, Bremen, Germany). Five microlitres of each sample were separated using a Kinesis KX Syringe Filter PTFE 13 mm pore size 0.45 µm (Kinesis Scientific Expert, Cole Parmer, St. Neots, UK). Three biological replications were used in the analysis. A water gradient containing formic acid (A, LC-MS grade, Merck Millipore, Burlington, MA, USA) and an acetonitrile gradient (B, LC-MS grade, Merck Millipore, Burlington, MA, USA) were employed as follows: a gradient of 5–75% B was applied from 1 to 14 min; a gradient of 75–99% B was used from 14 to 16 min; an isocratic condition of 99% B was maintained from 16 to 19 min; the gradient returned to 5% B from 19 to 20 min. The flow rate was maintained at 400 µL min^−1^. The columns were balanced using a 5% B solution for 3 min prior to analysis. A photodiode array (PDA) detector captured ultraviolet–visible (UV-Vis) spectra within the wavelength range of 250 to 550 nanometres at a frequency of 20 Hertz. An HESI-II (in the negative ion mode) had a spray voltage of −3.5 kV and an ion transfer tube temperature of 350 °C. Nitrogen was utilized as a sheath, auxiliary, and sweep gas at flow rates of 35, 10, and 3 arbitrary units, respectively. The auxiliary gas temperature was set to 400 °C, while the S-lens rf level was adjusted to 50. The FullMS scans were obtained throughout the 120–1800 *m*/*z* range with a resolution of 70,000 FWHM and a maximum ion trap time of 200 ms. The ddMS2 scans, with a TopN value of 5, were registered at a resolution of 17,500 FWHM and a maximum ion trap time of 100 ms. The raw files were pre-processed using MS-DIAL version 5.5 for peak selection and annotation using MSMS_Public_ExpBioInsilico_NEG_VS19 [23,24].

### 2.7. Cell Line and Culture Methods

The cell lines, MRC-5 and MeWo, were purchased from the American Type Cell Culture Collection (ATCC^®^, Manassas, VA, USA). The human fetal lung fibroblast cell line MRC-5 (ATCC^®^ CCL-171^TM^) was cultured in Eagle’s Minimum Essential Medium (EMEM; Biowest, Nuaillé, France) with addition of 1% (*v*/*v*) nonessential amino acids (Sigma-Aldrich, Burghausen, Germany) and 10% (*v*/*v*) FBS (Sigma-Aldrich, Burghausen, Germany). The malignant melanoma cell line MeWo (ATCC^®^ HTB-65™) was maintained in an EMEM medium with 10% (*v*/*v*) FBS (Sigma-Aldrich, Burghausen, Germany) supplementation. The cells were grown to near confluence in 100 × 15 mm cell culture Falcon^®^ Petri dishes (Corning, Warsaw, Poland) in a humidified chamber with maintaining 90% (*v*/*v*) relative humidity at 37 °C and 5% CO_2_ (*v*/*v*) atmosphere. The morphology of cell cultures was observed using an Axiovert 40 CFL inverted microscope (Carl Zeiss, Poznan, Poland). The Mycoplasma Stain Kit (Lonza, Monteggio, Switzerland) was used periodically to verify the absence of mycoplasma.

### 2.8. Extract Preparation for MTT Assay

The dry *K. elatine* cell biomass was extracted three times for one hour each with 30 mL of 80% MeOH/H_2_O (80:20, *v*/*v*) using an ultrasonic bath at 80 °C. The methanolic extracts were subsequently evaporated to dryness at 40 ± 1 °C and redissolved in sterile distilled water to reach a 0.2 g/mL concentration. *K. elatine* fraction was prepared using solid-phase extraction Sep-Pak^®^ RP-18 microcolumns (Waters, Milford, MA, USA). The aqueous solution was administered to the microcolumn in 1 mL increments, followed by elution with 5 mL of sterile distilled water and 5 mL of newly prepared 40% MeOH/H_2_O (40:60, *v*/*v*) to obtain the 40% methanolic fractions. Cytotoxic activity of the methanol extract and 40% methanol fraction from the cell biomass were evaluated on MeWo and MRC-5 cell lines using MTT assay.

### 2.9. MTT Assay

The tested cells were cultured at 5 × 10^3^ density into each well of a sterile 96-well plate in 100 μL/well medium and incubated in a humidified chamber with controlled conditions as described previously. KE-Ex, KE- Fr 40%, and acteoside (as a reference compound) were used to treat both cell lines in a 0–800 μg/mL concentration range. Dimethylsulfoxide (DMSO) was used to dissolve all extracts and fractions, with a final solvent concentration of 0.25% *v*/*v*. The extract and fraction were exposed to both cell lines for 24, 48, and 72 h. Each well was filled with 5 mg/mL of MTT (3-(4,5-dimethylthiazol-2-yl)-2,5-diphenyltetrazolium bromide) solution (Sigma-Aldrich, Schnelldorf, Germany) and incubated for an additional four hours at 37 °C. Next, 100 μL of solubilization buffer (10% SDS in 0.01 M HCl) was added. The absorbance at 570 nm was measured using Microplate Reader Multiscan FC (Thermo Scientific, Waltham, MA, USA) with a reference wavelength of 690 nm. Three separate experiments were carried out for each concentration, each with three repeats. The formula used to assess the relative cell viability is as follows:

$$\%\;cell\;viability=\frac{mean\;ofA570-A690\;of\;the\;experimental\;group}{mean\;ofA570-A690\;of\;the\;control\;group}\times 100\%$$

Excel software (Microsoft, Redmond, WA, USA) was used to calculate the viability of cells. The IC_50_ values are given as an estimate ± SE and were derived using the nonlinear regression program CompuSyn version 2022 (ComboSyn Inc., Paramus, NJ, USA) [25].

### 2.10. Statistical Analysis

The gathered data for metabolomic analyses were subjected to a one-way analysis of variance (ANOVA) as well as Duncan’s post hoc test. The results are reported as mean ± SE (standard error), with a two-sided *p*-value of 0.05 applied to determine the statistical significance. The obtained data from the MTT assay were expressed as mean ± SD from three independent experiments. Jamovi was used for statistical analyses (The jamovi project (2024). jamovi (Version 2.5) [Computer Software]. Retrieved from https://www.jamovi.org, accessed on 13 March 2025). ANOVA and Tukey post hoc test were used to assess the *p*-value. Furthermore, the MetaboAnalyst 6.0 web platform (http://www.metaboanalyst.ca/, accessed on 11 October 2024) was used to perform statistical analyses of the HRMS-MS data. Pareto scaling was used to normalize data, and a Partial Least Squares Discriminant Analysis (PLS-DA) plot was generated using three components.

## 3. Results

### 3.1. Callus Induction and Proliferation

**Figure 1:**
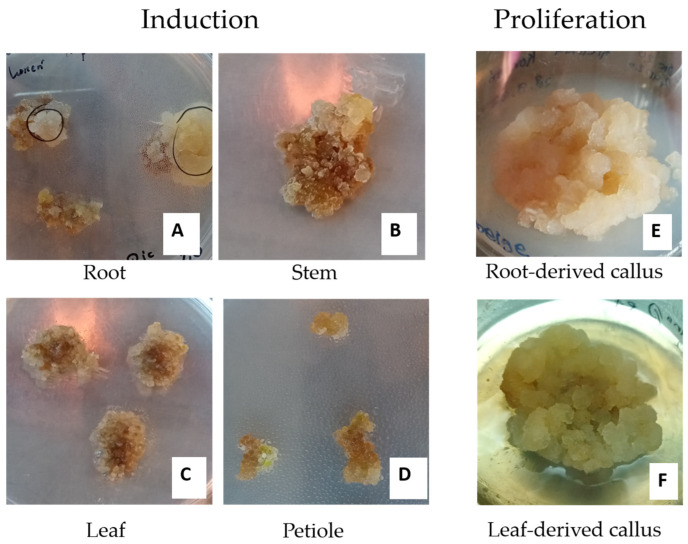
*Kickxia elatine* (L.) Dumort callus induction from root (**A**), stem (**B**), leaf (**C**), and petiole (**D**) explants. Proliferation of root-derived *K. elatine* callus on MS media with 2.0 mg L^−1^ Dic + 2.0 mg L^−1^ 2,4-D (**E**) and the leaf-derived callus on MS supplemented with 2.0 mg L^−1^ Dic + 0.5 mg L^−1^ 2,4-D (**F**).

This study remarks on the introduction of *K. elatine* (L.) Dumort into in vitro condition for the first time. The obtained results demonstrated the successful induction of callus from different explants of *K. elatine.* The callus induction was initiated from the fragments of roots, stems, petioles, and leaves of in vitro-derived culture (Table 1, Figure 1). The plant tissue samples were placed in five different types of media. Each media contained various amounts of Dic 2,4-D, along with a specific combination of 1.0 mg L^−1^ Dic + 0.1 mg L^−1^ TDZ. Callus formation occurred within a period of 14 to 21 days after the explant was put on the media. The data were gathered after six weeks and are presented in Table 1. Afterwards, the callus cultures were transferred into a medium with the same hormonal composition.

The leaves and petioles explants demonstrated the highest rate of callus formation. All of the explants reacted, resulting in a 100% response rate. Calluses formed at the wound sites. When cultivated on media with different concentrations of Dic and 2,4-D, the calluses exhibited a clumped, yellow-beige appearance (Figure 1). Callus formation was observed on all exposed petioles, but the reaction was relatively modest. Homogeneous, friable, and yellow-beige calluses were the most common morphology across different explants. Some explants, particularly petioles and leaves, induced compact calluses with high organogenesis when cultured with TDZ. High concentrations of Dic 2.0 mg mL^−1^ and 2,4-D 2.0 mg mL^−1^ generally resulted in friable and watery callus formations. The combination of Dic and TDZ resulted in the best proliferation, as well as compact and heterogeneous callus morphology. The results suggest a significant influence of TDZ on callus structure. The root and stem explants exhibited the maximum response rate (100%) when cultured on a medium supplemented with 1.0 mg L^−1^ Dic and 0.1 mg L^−1^ TDZ, resulting in good proliferation of cells. However, the callus was heterogeneous, clumpy, and organogenic.

The growth index of *K. elatine* callus cultures derived from the root and leaf explants was calculated and is presented in Table 2 below. The growth index of *K. elatine* callus cultures cultivated on MS supplemented with 2.0 mg L^−1^ Dic and 2.0 mg L^−1^ 2,4-D was 351.71 ± 27.77. Correspondingly, the calluses derived from the MS medium containing 2.0 mg L^−1^ Dic and 0.5 mg L^−1^ 2,4-D exhibited a growth index of 288.05 ± 42.60. Both calluses were homogeneous in beige colour, with clumpy and watery characteristics. The root-derived calluses were the better option for initiating a suspension culture due to their higher proliferation rate, homogeneous morphology, and friable structure.

### 3.2. Cell Suspension Culture

**Figure 2:**
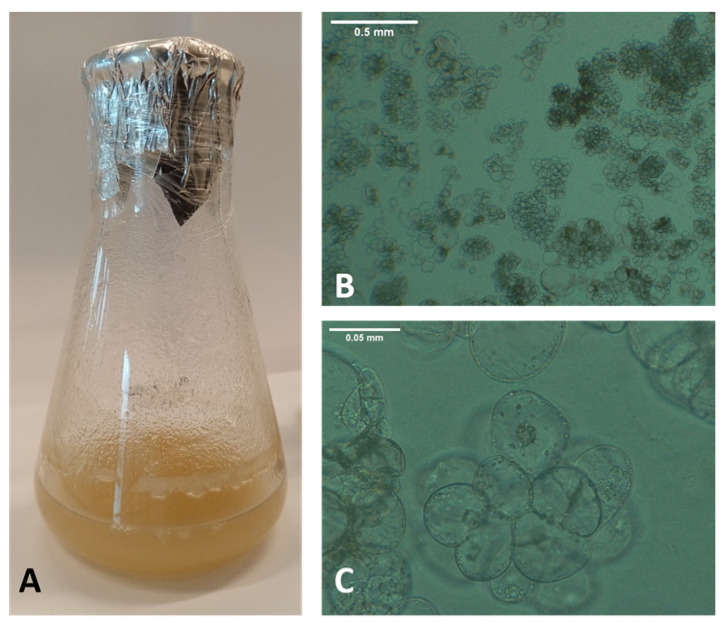
Cell suspension culture of *Kickxia elatine* (L.) Dumort (passage 6), established from root-derived callus, grown in MS 2.0 mg L^−1^ Dic and 2.0 mg L^−1^ 2,4-D and agitated at 110 RPM in a controlled room with a 16:8-hour photoperiod at 20 ± 2 °C (**A**). The microscopic view of *K. elatine* cell culture (day 18) shows clusters of small and round cells on observation using 50× (**B**) and 400× (**C**) magnification.

The cell suspension culture of *K. elatine* was established from root-derived callus cultures and maintained in MS 2.0 mg L^−1^ Dic and 2.0 mg L^−1^ 2,4-D (Figure 2A). Cell viability, aggregation of cells, and risk of contamination were observed under a microscope every three days of culture. The suspension cultures of *K. elatine* cells were characterized by round or oval shapes in various sizes and a tendency to aggregate (Figure 2B,C). Furthermore, intense cell divisions could be observed in the suspension cells (Figure 2B).

**Figure 3:**
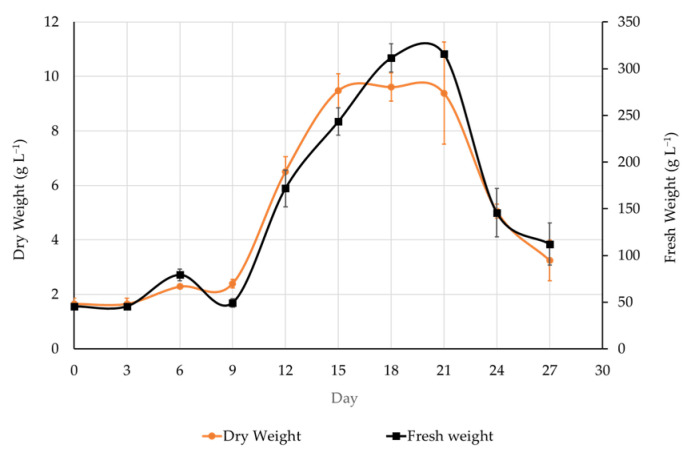
Time course of fresh (right *y*-axis) and dry weight (left *y*-axis) biomass accumulation of *Kickxia elatine* (L.) Dumort cell suspension culture system in 30 mL Murashige and Skoog liquid media supplemented with 2.0 mg L^−1^ Dic + 2.0 mg L^−1^ 2,4-D and 3% sucrose (D). The mean of fresh (black line) and dry weight (orange line) was obtained from three biological replications per observation time.

In this study, the growth curve of *K. elatine* cell culture in 2.0 mg L^−1^ Dic + 2.0 mg L^−1^ 2,4-D was observed. As the results were similar, the growth kinetic curve of the fresh and dry weight biomass of *K. elatine* cell suspension culture presented in Figure 3 represents the overall result. The lag phase was observed until day 9 of the culture, followed by the log phase, which started from day 9 and continued until day 18. The stationary phase of *K. elatine* cell culture in this media was notably brief, spanning from day 18 to day 21. Subsequently, the cell biomass was seen to decrease, possibly due to the senescence of the cells. During the cell culture period of 27 days, the highest fresh and dry weights were obtained from cells cultured in 2.0 mg L^−1^ Dic + 2.0 mg L^−1^ 2,4-D, with 9.48 g and 0.28 g (day 21), respectively. Therefore, to maximize the biomass yield, subculturing at day 15–20 is recommended before the decline phase begins (Figure 3). The observed growth pattern suggests that *K. elatine* cells successfully adapted to liquid culture conditions.

### 3.3. Nuclear DNA Content

**Figure 4:**
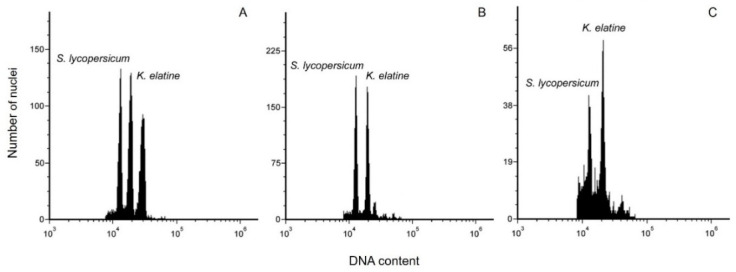
Histograms of nuclear DNA content obtained using flow cytometric analysis of the PI-stained nuclei isolated simultaneously from the leaves of *Solanum lycopersicum* L. (internal standard) and *Kickxia elatine* (L.) Dumort: (**A**) seeds, (**B**) in vitro-derived shoot passage 5, and (**C**) cell suspension culture passage 3.

Flow cytometry assay controls the genetic fidelity of in vitro-derived plant material, which may be altered by somaclonal variation [26]. In this study, the flow cytometric analysis from seeds, in vitro-derived shoots, and cell suspension culture revealed that the nuclear DNA content was 2.720 ± 0.111, 2.916 ± 0.021 and 3.165 ± 0.017 pg/2C, respectively (Table 3 and Figure 4). The measurements revealed low diversity between the investigated plant materials. However, this outcome may result from the age of callus culture or high concentration of secondary metabolites, which might disturb the DNA staining process and lead to small differences in DNA content.

### 3.4. UPLC-HRMS/MS Analysis

**Figure 5:**
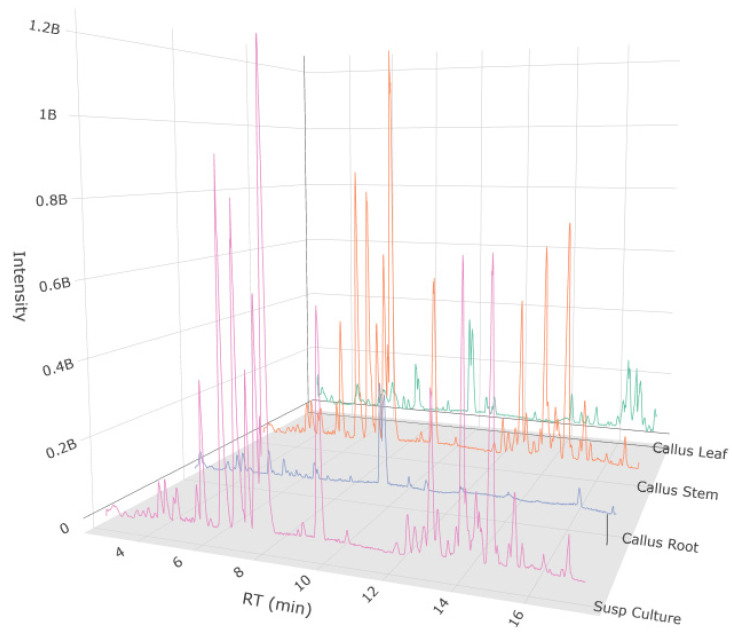
Base peak chromatogram of *Kickxia elatine* (L.) Dumort calluses derived from leaf (green), stem (orange), root (blue) explants, and cell suspension culture (red) from UPLC-HRMS/MS in negative ion mode.

The metabolites of *K. elatine* callus and cell suspension cultures were analyzed and annotated using an LC-MS/MS approach (Figure 5). The untargeted analyses revealed the presence of amino acids and derivatives, medium- and long-chain fatty acids along with derivatives, and carboxylic acids. Selected metabolites, which were identified in this study from the *K. elatine* callus and cell suspension cultures, are listed in Table 4 (full annotated compounds are available in Table S1). According to the tentative annotation of metabolites, a broad panel of secondary metabolites was detected in the extracts of callus and cell suspensions. The major detected groups belong to benzoic acid derivatives, phenolic glycosides, phenylpropanoic acids, hydroxycinnamic acid derivatives, and tyrosol derivatives.

**Figure 6:**
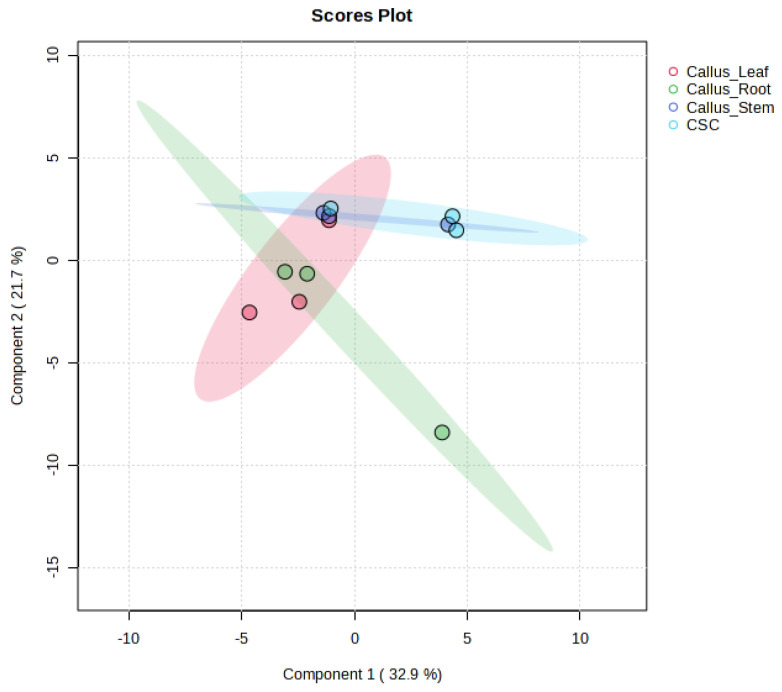
PLS-DA score plot was derived from UPLC-HRMS/MS data of secondary metabolites extracted from *Kickxia elatine* (L.) Dumort callus initiated from leaf (red), root (green), and stem (blue), and cell suspension culture (light blue).

The obtained data (three replications) were subjected to log10 transformation and partial least squares discriminant analysis (PLS-DA), as presented in Figure 6. PLS-DA analysis of the metabolite profile from callus and cell suspension culture of *K. elatine* did not result in a robust model, suggesting that the metabolite profiles were not significantly different to distinguish between the samples.

### 3.5. Cytotoxic Assay Using MTT

**Figure 7:**
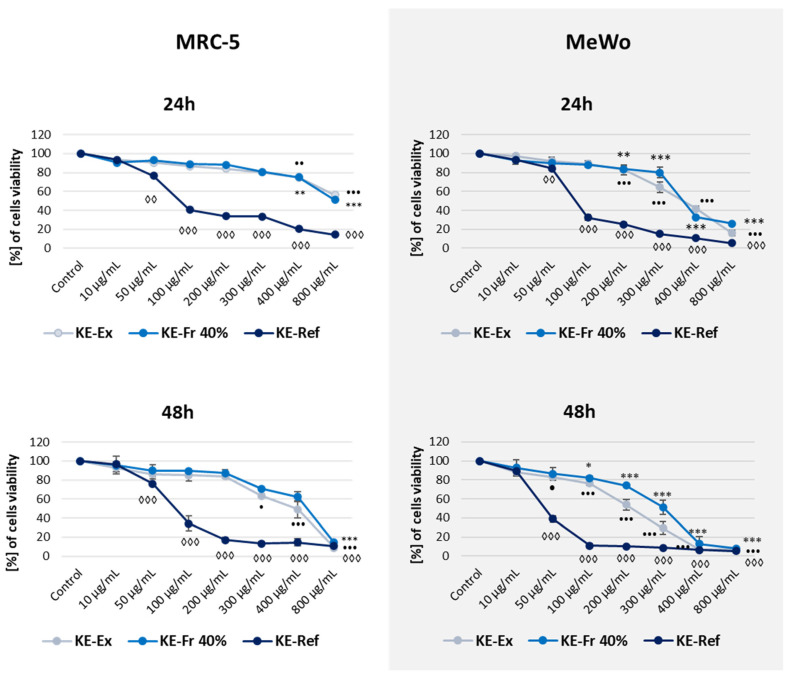

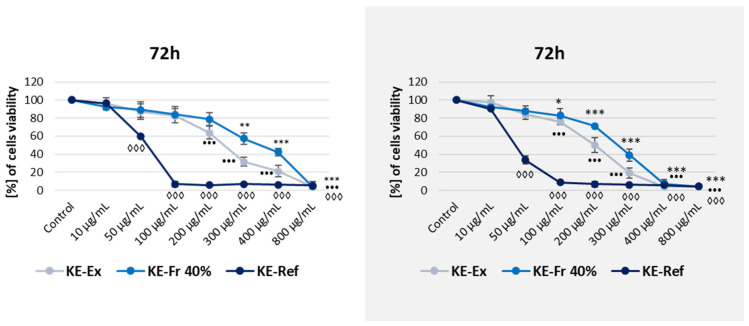
The viability assessment of human fibroblast MRC-5 and human melanoma MeWo cells after treatment with methanolic extracts (KE-Ex), 40% MeOH fraction (KE-Fr 40%), and acteoside (KE-Ref) from *Kickxia elatine* (L.) Dumort callus biomass for 24, 48, and 72 h presented as a line graph. The cell viability is expressed as a percentage of the nontreated control cells. The mean of three experiments ± SD is shown. Symbols of respective levels of significance (*,•, for *p* < 0.05; (**, ••, ◊◊) for *p* < 0.01; (***, •••, ◊◊◊) for *p* < 0.001.

The cytotoxic activity of the extract and methanolic fractions of *K. elatine* callus biomass was evaluated against the human melanoma (MeWo) and noncancerous human lung fibroblast (MRC-5) cell lines using the MTT test. The extract and fraction were administered to both cell lines at concentrations ranging from 0 to 800 μg mL^−1^ for 24, 48, and 72 h. Acteoside was chosen as the reference due to prior findings indicating its presence in *K. elatine* and within the plant cell culture, as shown by the LC-MS result in this study. As shown in Figure 7, the results are expressed as a percentage of cell number compared to the untreated samples. Acteoside, the reference compound (KE-Ref), showed potent toxicity and decreased the MeWo and MRC-5 cell viability by over 60% and 90% at 100 μg mL^−1^ depending on observation times, respectively. The cell viability evaluation of KE-Ex and KE-Fr 40% was similar in lower concentrations of compounds; however, it showed selective cytotoxicity toward the MeWo melanoma cell lines and worked in a dose- and time-dependent manner. A significant decrease in cell proliferation (around 20%) was shown in the concentration of 200 µg mL^−1^ for 24 h in MeWo cells. A longer time of incubation (48 and 72 h) with KE-Ex and KE-Fr 40% reveals a reduction of cell viability in a lower concentration of tested compounds (100 μg mL^−1^). However, for the same decrease (20%) in the viability of normal fibroblast, MRC-5 cells were observed in higher KE-Ex and KE-Fr 40% concentrations (respectively, 400 µg/mL and 200 µg mL^−1^). Furthermore, the results show that *K. elatine* cell extract has more potential in both studied cell lines compared to its 40% methanolic fractions for its cytotoxic activity.

The half-maximal inhibitory concentration (IC_50_) value is a standard method for determining the cytotoxicity level, as it indicates the concentration of tested samples capable of killing 50% of the cells. The KE-Ex and KE-Fr 40% showed low cytotoxicity against the MeWo cell line after treatment for 24 h, with IC_50_ values of 345 ± 5 μg mL^−1^ and 340 ± 15 μg mL^−1^, respectively. Noteworthy, KE-Ex and KE-Fr 40%, in the treatment time of 24 h, did not influence the viability of normal fibroblasts, MRC-5 cell line, with IC_50_ value over 800 µg mL^−1^. The IC_50_ value of the reference compound, acteoside, in the MeWo cell line reached 75 ± 7 µg mL^−1^ after 24 h of treatment and in the MRC-5 cell line achieved 105 ± 1 μg mL^−1^ (Table 5). The MTT results confirm the in vitro selectivity of the extract and 40% methanol fractions cytotoxicity against melanoma MeWo cell line.

## 4. Discussion

The present study successfully initiated the callus culture of *K. elatine* from the petiole, stem, leaf, and root on an MS medium supplemented with 2,4-D and Dic in various concentrations and on MS media added to Dic and TDZ. The effectiveness of 2,4-D (with or without combining other growth regulators) for callus initiation has been recorded from the Plantagianceae family, notably *Plantago camtschatica* [27], *P. lanceolata* [28], *P. ovata* [29,30], and *Digitalis lanata* [31]. Rahamooz-Haghighi et al. [32] observed the induction of *P. lanceolata* callus in the presence of thidiazuron with or without combination with auxins. Their results suggest that thidiazuron works best when coupled with auxins (ranging between 20% and 100%). Accordingly, the data from this study present a similar conclusion. The biomass production of *K. elatine* cells is reported in this study for the first time.

Somaclonal variants include genetic variability from in vitro culture techniques, particularly in cell cultures. Prolonged culture time, high concentration of growth regulators, and in vitro culture conditions are among the factors that promote instability in cell cultures [33]. It is hypothesized that the cell cultures obtained in this study were undergoing somaclonal variations, as suggested by the results shown in Table 3 and Figure 4. Previous studies on the genus Plantaginaceae reported similar findings [26,34]. Makowczyńska and Sliwinska [26] observed lowered 2C DNA content of *Plantago asiatica* L. in vitro-derived leaves (3.296 ± 0.054 2C/pg) compared to nonorganogenic calluses (2.971–3.451 2C/pg). Kour et al. [34] observed genetic variation in *P. lagopus* L. by SSAP and MSAP analyses. They postulated that recombination and transposable elements were responsible for the genetic variation event. Furthermore, their findings indicated potential increases and decreases in DNA methylation occurrences in in vitro-regenerated *P. lagopus*. Moreover, the supplementation of 2,4-D could also induce genetic damage or epigenetic changes. The negative impacts of 2,4-D on callus have been previously observed in *Coffea arabica* and *C. canephora* [35]. These data offer an overview of the possible causes influencing variation in our current investigation. Interestingly, nuclear DNA content from *K. elatine* seeds (control) evaluated in this research is generally higher than other reported *Kickxia* species, *K. spuria* and *K. scoaria*, with 1.64 and 1.87 2C/pg, respectively [36,37]. This is the first recording of *K. elatine* nuclear DNA content using flow cytometry. The observed alterations in DNA content and genetic variation support the concept that somaclonal variation significantly contributed to the genetic instability noted in the cultures.

The application of plant biotechnology to obtain cell biomass for medical purposes has been advancing in recent years. Daphnenone, daphnolon, R-(−)-1-(4′-hydroxyphenyl)-3-hydroxy-5-phenyl-1,5-pentandione, and S-(+)-daphneolone-4′-O-β-d-glucoside from *Daphne giraldii* callus cells showed cytotoxicity to the human melanoma A375-S2 cell line, with IC_50_ values 29.8, 51.0, 41.0, and 150.0 μM, respectively [38]. *Silybum marianum* L. cell culture extract (15–125 μg mL^−1^) was observed to potentially inhibit pro-inflammatory cytokine production in skin cells, thus presenting a promising skin cancer preventive and therapeutic agent [39].

The main treatment strategy for melanoma is surgery and radiotherapy, especially in the early stages of the disease. However, more advanced stages of cancer require targeted cancer drugs, chemotherapy, and immunotherapy. Dacarbazine (DITC) has been the FDA-approved first-line treatment for advanced metastatic melanoma in routine clinical practice for over 30 years. DTIC methylates nucleic acids, causing DNA damage resulting in growth arrest and cell death. However, as results show, long-term DITC treatment has low-range responses (around 5–10% of patients) and causes side effects, especially gastrointestinal, but also anemia, neutropenia, or diarrhea [40]. The low therapeutic efficacy of dacarbazine might be connected with the rapid removal of DNA lesions by repair systems [41]. The IC_50_ value of DITC for 48 h in MeWo cells is 430 μg mL^−1^ [42].

The real breakthrough in melanoma patients’ treatment with metastasis was the biological activity of monoclonal antibodies working as immunomodulators, like ipilimumab or nivolumab, blocking the repressor of T lymphocyte activation (CTLA-4) and programmed cell death protein (PD-1). Ipilimumab, together with nivolumab, significantly increases the progression-free survival of patients with metastatic melanoma [43]. Other drugs, like vemurafenib, dabrafenib, and encorafenib, which are BRAF inhibitors, were approved by the FDA for the treatment of melanoma with the BRAF V600 mutation [44]. However, because of the development of these drugs’ resistance, combination therapies with MEK (MAPK/ERK kinase) inhibitors (MEKi) have been applied to delay or prevent the resistance [42].

Only a few studies have documented anticancer activities in *Kickxia* species. The isolated 6,7-dimethoxy-5-hydroxyflavone from *K. spuria* could inhibit the proliferation activity of human cervix carcinoma HeLa cell lines when observed using the xCELLigence assay [45]. Additionally, flavonoids from *K. aegyptiaca* showed promising cytotoxic activity against breast (MCF-7) and colon (HCT-116) cell lines. Pectolinarigenin isolated from this species was highly toxic, with 91.1% and 37.9% toxicity against MCF-7 and HCT-116 at 100 µg mL^−1^, respectively [46]. In this study, the cytotoxicity potential of methanolic extract and the fraction of *K. elatine* cell biomass, along with acteoside, was assessed against MeWo (malignant melanoma cell line) and MRC-5 (human fetal lung fibroblast cell line) using the MTT assay. Our results reveal the selective biological activity of the KE-Ex and KE-Fr 40% toward the MeWo melanoma cell line.

The cytotoxic activity of *K. elatine* cell biomass extract against melanoma (MeWo) cells demonstrated a dose- and time-dependent effect. A similar result was observed in a 2024 study on *E. planum* and *L. flos-cuculi*. However, key differences lay in the IC_50_ values, selectivity, and active compounds, highlighting variations in their anticancer potential. *K. elatine* extract exhibited moderate toxicity against MeWO cells, with an IC_50_ value more than double that of *E. planum* and *L. flos-cuculi.* The strong activity of both species can be attributed to triterpenoid saponins, which were not detected in *K. elatine* [47].

The cytotoxicity of *K. elatine* against melanoma cells may be related to the compounds present in the biomass, mostly fatty acids and their derivatives, linoleic acids, and their derivatives, and notably acteoside. Acteoside presence is reported in the cell culture of *K. elatine* (this study; Table 4). Anticarcinogenic activity of acteoside has been examined in various cancer cell lines previously [48,49,50,51]. Several studies have studied the mechanism of acteoside against melanoma cell lines. Cheimonidi et al. [52] reported that acteoside isolated from *Lippia citriodora* (Paláu) Kunth (syn. *Aloysia citrodora* Paláu) enhanced reactive oxidative stress (ROS) levels, which activated antioxidant and proteostasis mechanisms in the mouse melanoma cell lines B16.F1 and B16.F10. Moreover, using the melanoma model of C57BL/6 mice with grafted B16.F1 cells, acteoside was found to affect oncogenic pathways via protein kinase C inhibition. Furthermore, acteoside inhibited the growth and development and promoted apoptosis of melanoma cells via regulation of the ERβ-Ras/Raf1-STAT3 signalling axis in BALB/C nude mice [53].

The presence of fatty acids, which were dominant in the extract of *K. elatine* cell biomass, may enhance its cytotoxicity against melanoma cells. As listed in Table 5 and Table S1, linoleic acid and its derivatives were abundant in *K. elatine* cell extracts. Early studies suggested that vegetable oils rich in linoleic acid (3–100 µg mL^−1^) demonstrated selective antineoplastic properties. Sesame oils, which contain high levels of linoleic acid, selectively inhibited malignant SK-MEL melanoma cell growth compared to NHEM normal human epidermal melanocytes [54]. Further studies revealed the mechanism of action of linoleic acids on melanoma cells. According to De Sousa Andrade et al. [55], linoleic acid was highly toxic to the human SK-Mel 23 cell line at 200 µM via the loss of membrane integrity, causing the melanoma cells to undergo relative size reduction and a significant increase in cell granularity after 48 h of treatment. The toxicity of linoleic acid was postulated to work through metabolic derivatives or neutral lipid accumulation in the cytosol or mitochondrial depolarization. Ando et al. proposed that linoleic acid decreased melanin synthesis by accelerating the proteolytic degradation of tyrosinase through the ubiquitin proteasome pathway [56,57,58]. Studies on the impact of linoleic acid, or linoleic-containing plant oil, against the B16-F10 murine melanoma cell line are in agreement with the previous results. Linoleic acid or linoleic-containing plant oil affects the trafficking of tyrosine, resulting in the suppression of melanogenesis [59,60,61].

## 5. Conclusions

The present study demonstrated successful cell culture initiation and maintenance, which opens up the possibility of its biomass application and metabolite production. The metabolite profiles are similar between the callus and cell suspension culture of *K. elatine*, consisting of benzoic acid derivatives, phenolic glycosides, phenylpropanoic acids, hydroxycinnamic acid derivatives and tyrosol derivatives, amino acids, peptides, and fatty acids. Preliminary cytotoxic assays confirmed that *K. elatine* cell biomass extract exhibited significant antiproliferative activity against the MeWo melanoma cell line with dose- and time-dependent effects. To further optimize bioactive compound production, elicitation strategies should be integrated into future research. The findings of this study highlight the biotechnology potential of *K. elatine* as a valuable source of cytotoxic metabolites, contributing to the development of plant-derived anticancer therapeutics.

## Figures and Tables

**Table 1:** Induction and morphological characteristics of *Kickxia elatine* (L.) Dumort callus.

Type of Explant	Plant Growth Regulators (mg L^−1^)			Induction Percentage (%)	Proliferation *	Morphology
	Dic	2,4-D	TDZ			
Roots	2.0	0.5	-	85.7	+++	Heterogeneous, friable, yellow-beige and green colour
	2.0	1.0	-	100	++	Homogeneous, friable, watery, yellow-beige colour
	2.0	2.0	-	100	+	Homogeneous, friable, watery, beige colour
	1.0	2.0	-	100	+	Homogeneous, friable, watery, light beige colour
	1.0	1.0	-	92.9	++	Heterogeneous, friable, yellow-beige and green colour
	1.0	-	0.1	100	++++	Heterogeneous, clumpy and compact, beige and green colour
Stems	2.0	0.5	-	70	+	Homogeneous, friable, yellow-beige colour
	2.0	1.0	-	60	+	Homogeneous, friable, yellow-beige colour
	2.0	2.0	-	86.7	+	Homogeneous, friable, yellow-beige colour
	1.0	2.0	-	70	+	Homogeneous, friable, yellow-beige colour
	1.0	1.0	-	40	+	Homogeneous, friable, yellow and beige colour
	1.0	-	0.1	100	++++	Heterogeneous, clumpy and compact, beige and green colour, organogenesis
Petioles	2.0	0.5	-	100	+	Homogeneous, friable, yellow-beige colour
	2.0	1.0	-	100	+	Homogeneous, friable, yellow-beige colour
	2.0	2.0	-	100	+	Homogeneous, friable, yellow-beige colour
	1.0	2.0	-	100	+	Homogeneous, friable, yellow-beige colour
	1.0	1.0	-	100	++	Homogeneous, friable, yellow-beige colour
	1.0	-	0.1	100	+++	Heterogeneous, clumpy and compact, yellow-beige colour, organogenesis
Leaves	2.0	0.5	-	100	+++	Homogeneous, friable, yellow-beige colour
	2.0	1.0	-	100	+++	Homogeneous, friable, yellow-beige colour
	2.0	2.0	-	100	++	Homogeneous, friable, yellow-beige colour
	1.0	2.0	-	100	++	Homogeneous, friable, cream colour
	1.0	1.0	-	100	++	Homogeneous, friable, cream colour
	1.0	-	0.1	100	++++	Homogeneous, clumpy and compact, organogenesis

* Proliferation: - not proliferating, + poor, ++ weak, +++ good, ++++ best. Abbreviation: Dic—Dicamba; 2,4-D—2,4-dichlorophenoxyacetic acid; TDZ—Thidiazuron.

**Table 2:** Growth parameters of *Kickxia elatine* (L.) Dumort calluses derived from root and leaf explants on MS medium supplemented with Dic + 2,4-D.

		Callus Growth Index (%) ± SE	Mean	*p*-Value
2.0 mg L^−1^ Dic 2.0 mg L^−1^ 2,4-D (root-derived)	Passage 5	418.83 ± 36.41	351.71 ± 27.77	0.286
	Passage 6	272.81 ± 46.75		
	Passage 7	303.31 ± 28.01		
	Passage 8	398.25 ± 27.97		
	Passage 9	365.33 ± 41.79		
2.0 mg L^−1^ Dic 0.5 mg L^−1^ 2,4-D (leaf-derived)	Passage 4	273.65 ± 9.97	288.05 ± 42.60	
	Passage 5	267.51 ± 25.70		
	Passage 6	406.86 ± 34.55		
	Passage 7	204.19 ± 13.77		

Abbreviation: Dic—Dicamba; 2,4-D—2,4-dichlorophenoxyacetic acid. *p*-value significant if <0.05.

**Table 3:** Nuclear DNA content in leaves from the seeds, in vitro-derived shoots, and cell cultures of *Kickxia elatine* (L.) Dumort.

Plant Material	DNA Content (pg/2C ± SD)
Seeds	2.720 ± 0.111 c *
In vitro-derived shoot passage 5	2.916 ± 0.021 b
Cell suspension culture passage 3	3.165 ± 0.017 a

*—Mean values within a column followed by the same letter are not significantly different at *p* = 0.05 (Duncan’s test).

**Table 4:** Selected identified metabolites in callus and cell suspension of *Kickxia elatine* (L.) Dumort annotated using MS DIAL 5.5.

No	RT (min)	Formula	Adduct Type	Measured *m*/*z*	Reference *m*/*z*	Metabolite Name	Ontology
1	2.78	C_9_H_11_NO_2_	[M-H]-	164.0704	164.0717	Phenylalanine	Phenylalanine and derivatives
2	3.22	C_9_H_17_NO_5_	[M-H]-	218.1029	218.1034	Pantothenate	Secondary alcohols
3	3.89	C_11_H_12_N_2_O_2_	[M-H]-	203.0818	203.0826	Tryptophan	Indolyl carboxylic acids and derivatives
4	4.50	C_7_H_12_O_5_	[M-H]-	175.06	175.0612	2-Isopropylmalic acid	Hydroxy fatty acids
5	4.64	C_19_H_28_O_11_	[M-H]-	431.1556	431.1559	(2R,3S,4S,5R,6R)-5-[(2S,3R,4R)-3,4-dihydroxy-4-(hydroxymethyl)oxolan-2-yl]oxy-2-(hydroxymethyl)-6-[2-(4-hydroxyphenyl)ethoxy]oxane-3,4-diol	Phenylpropanoids
6	5.32	C_9_H_8_O_4_	[M-H]-	179.034	179.035	Caffeic acid	Hydroxycinnamic acids
7	5.84	C_19_H_28_O_10_	[M-H]-	415.1614	415.161	(2R,3S,4S,5R,6R)-2-[[(2S,3R,4R)-3,4-dihydroxy-4-(hydroxymethyl)oxolan-2-yl]oxymethyl]-6-(2-phenylethoxy)oxane-3,4,5-triol	Phenylpropanoids
8	6.19	C_35_H_46_O_20_	[M-H]-	785.2514	785.251	Echinacoside	Phenylpropanoids
9	6.31	C_34_H_44_O_19_	[M-H]-	755.2402	755.2404	Lavandulifolioside	Phenylpropanoids
10	6.67	C_29_H_36_O_15_	[M-H]-	623.1978	623.1981	Acteoside	Phenylpropanoids
11	6.71	C_10_H_18_O_5_	[M-H]-	217.1074	217.1082	Hydroxysebacic acid	Medium-chain hydroxy acids and derivatives
12	6.92	C_9_H_8_O_3_	[M-H]-	163.0389	163.0401	trans-4-Coumaric acid	Hydroxycinnamic acids
13	7.09	C_29_H_36_O_15_	[M-H]-	623.1979	623.1981	Isoacteoside	Phenylpropanoids
14	7.19	C_30_H_37_O_15_	[M-H]-	637.2136	637.2127	((2R,3R,4R,5R,6R)-6-(2-(3,4-dihydroxyphenyl)ethoxy)-5-hydroxy-2-(hydroxymethyl)-4-((2S,3R,4R,5R,6S)-3,4,5-trihydroxy-6-methyloxan-2-yl)oxyoxan-3-yl) (E)-3-(4-hydroxy-3-methoxyphenyl)prop-2-enoate	Phenylpropanoids
15	7.39	C_9_H_16_O_4_	[M-H]-	187.0965	187.0976	Azelaic acid	Medium-chain fatty acids
16	9.17	C_18_H_34_O_5_	[M-H]-	329.2335	329.2333	(Z)-5,8,11-trihydroxyoctadec-9-enoic acid	Long-chain fatty acids
17	10.61	C_18_H_30_O_4_	[M-H]-	309.2074	309.2071	FA 18:3 + 2O	Linoleic acids and derivatives
18	14.10	C_18_H_32_O_3_	[M-H]-	295.2277	295.2279	9-HODE	Linoleic acids and derivatives
19	14.77	C_18_H_34_O_3_	[M-H]-	297.2434	297.2435	FA 18:1 + 1O	Linoleic acids and derivatives
20	14.91	C_18_H_32_O_2_	[M-H]-	279.2327	279.2329	Linoleic acid	Linoleic acids and derivatives

**Table 5:** The effect of methanolic extract and 40% methanolic fractions from *Kickxia elatine* (L.) Dumort cell biomass on MRC-5 and MeWo cell lines expressed as IC_50_ values. IC_50_ represents the concentration at which a substance exerts half its maximal inhibitory effect. Results were obtained from three independent experiments and presented as mean ± SE.

		IC_50_ [µg mL^−1^]		
Cell Line	Time	Extract	Fraction 40% MeOH	Reference (Acteoside)
MRC-5	24 h	>800	>800	105 ± 1
	48 h	268 ± 12	549 ± 5	79 ± 8
	72 h	166 ± 10	231 ± 12	35.5 ± 1
MeWo	24 h	345 ± 5	340 ± 15	75 ± 7
	48 h	125 ± 8	214 ± 12	35 ± 1
	72 h	117 ± 7	162 ± 10	22 ± 1

## Data Availability

The data are contained within the article or Supplementary Material.

## Supplementary Materials

The following supporting information can be downloaded at https://www.mdpi.com/article/10.3390/biomedicines13061382/s1. Table S1: Identified metabolites in callus and cell suspension culture of *Kickxia elatine* (L.) Dumort using MS-DIAL.

## References

[1] Dziankowska-Zaborszczyk E., Maniecka-Bryła I., Pikala M. (2022). Mortality Trends Due to Skin Melanoma in Poland in the Years 2000–2020. Int. J. Environ. Res. Public Health.

[2] Conforti C., Zalaudek I. (2021). Epidemiology and Risk Factors of Melanoma: A Review. Dermatol. Pract. Concept..

[3] Banik K., Ranaware A.M., Harsha C., Nitesh T., Girisa S., Deshpande V., Fan L., Nalawade S.P., Sethi G., Kunnumakkara A.B. (2020). Piceatannol: A Natural Stilbene for the Prevention and Treatment of Cancer. Pharmacol. Res..

[4] Bellosi B., Selldorf P., Schoenenberger N. (2011). Exploring the Flora on Inert Landfill Sites in Southern Ticino (Switzerland). Bauhinia.

[5] Węgrzynek B., Nowak T. (2010). Rare and Endangered Segetal Weed Species in the Silesian Upland (s Poland) Recorded in the Last Twenty Years. Plant Breed. Seed Sci..

[6] Unterladstetter V., Jagel A. (2018). Tännelkräuter—Zwei unauffällige Wildkräuter verlassen die Äcker. Palmengarten.

[7] Wren R.C. (1956). Potter’s New Cyclopaedia of Botanical Drugs and Preparations.

[8] Dhivya S.M., Kalaichelvi K. (2015). Studies on Ethno-Medicinal Plants Used by the Irulas Tribes of Nellithurai Beat, Karamadai Range of Western Ghats, Tamil Nadu, India. Int. J. Pharm. Chem. Sci..

[9] Janaćković P., Gavrilović M., Miletić M., Radulović M., Kolašinac S., Stevanović Z.D. (2022). Small Regions as Key Sources of Traditional Knowledge: A Quantitative Ethnobotanical Survey in the Central Balkans. J. Ethnobiol. Ethnomed..

[10] Yuldashev M.P., Malikov V.M., Batirov É.K. (1996). Flavonoids of the Epigeal Part of *Kickxia elatine*. Chem. Nat. Compd..

[11] Tuttolomondo T., Licata M., Leto C., Savo V., Bonsangue G., Letizia Gargano M., Venturella G., La Bella S. (2014). Ethnobotanical Investigation on Wild Medicinal Plants in the Monti Sicani Regional Park (Sicily, Italy). J. Ethnopharmacol..

[12] Toth L., Csordas I., Papay V. (1978). Chemical Analysis of *Kickxia elatine* (L.) Dum. Herba Hung..

[13] Toth L., Csordas I., Papay V., Bujtas G. (1978). The Flavonoids of *Kickxia elatine* (L.) Dum. Pharmazie.

[14] Handjieva N., Tersieva L., Popov S., Evstatieva L. (1995). Two Iridoid Glucosides, 5-O-Menthiafoloylkickxioside and Kickxin, from *Kickxia* Dum. Species. Phytochemistry.

[15] Efferth T. (2019). Biotechnology Applications of Plant Callus Cultures. Engineering.

[16] Hermosaningtyas A.A., Chanaj-Kaczmarek J., Kikowska M., Gornowicz-Porowska J., Budzianowska A., Pawlaczyk M. (2024). Potential of Plant Stem Cells as Helpful Agents for Skin Disorders—A Narrative Review. Appl. Sci..

[17] Wawrosch C., Zotchev S.B. (2021). Production of Bioactive Plant Secondary Metabolites through in Vitro Technologies—Status and Outlook. Appl. Microbiol. Biotechnol..

[18] Bapat V.A., Kavi Kishor P.B., Jalaja N., Jain S.M., Penna S. (2023). Plant Cell Cultures: Biofactories for the Production of Bioactive Compounds. Agronomy.

[19] Kikowska M., Thiem B., Nahorska A. (2015). Potential of Plant Cell Cultures as a Source of Bioactive Compounds for Cosmetic Applications (in Polish). Pol. J. Cosmetol..

[20] Niazian M., Sabbatini P. (2021). Traditional in Vitro Strategies for Sustainable Production of Bioactive Compounds and Manipulation of Metabolomic Profile in Medicinal, Aromatic and Ornamental Plants. Planta.

[21] Murashige T., Skoog F. (1962). A Revised Medium for Rapid Growth and Bioassays with Tobacco Tissue Cultures. Physiol. Plant..

[22] Kikowska M., Thiem B., Sliwinska E., Rewers M., Kowalczyk M., Stochmal A., Oleszek W. (2014). The Effect of Nutritional Factors and Plant Growth Regulators on Micropropagation and Production of Phenolic Acids and Saponins from Plantlets and Adventitious Root Cultures of *Eryngium maritimum* L.. J. Plant Growth Regul..

[23] Tsugawa H., Ikeda K., Takahashi M., Satoh A., Mori Y., Uchino H., Okahashi N., Yamada Y., Tada I., Bonini P. (2020). A Lipidome Atlas in MS-DIAL 4. Nat. Biotechnol..

[24] Tsugawa H., Nakabayashi R., Mori T., Yamada Y., Takahashi M., Rai A., Sugiyama R., Yamamoto H., Nakaya T., Yamazaki M. (2019). A Cheminformatics Approach to Characterize Metabolomes in Stable-Isotope-Labeled Organisms. Nat. Methods.

[25] Chou T.C., Martin N. (2005). CompuSyn for Drug Combinations: PC Software and User’s Guide: A Computer Program for Quantitation of Synergism and Antagonism in Drug Combinations, and the Determination of IC50 and ED50 and LD50 Values.

[26] Makowczyńska J., Andrzejewska-Golec E., Sliwinska E. (2008). Nuclear DNA Content in Different Plant Materials of *Plantago asiatica* L. Cultured in Vitro. Plant Cell Tissue Organ Cult..

[27] Ay N.V., Duy M.V., Baatartsogt O., Enkhchimeg V. (2017). Plant Regeneration of Kamchatic Plantain (*Plantago cantschatica* Link) through Callus Induction. Mong. J. Agric. Sci..

[28] Budzianowska A., Skrzypczak L., Budzianowski J. (2004). Phenylethanoid Glucosides from in vitro Propagated Plants and Callus Cultures of *Plantago lanceolata*. Planta Med..

[29] Wakhlu A.K., Barna K.S. (1989). Callus Initiation, Growth and Plant Regeneration in *Plantago ovata* Forsk. Cv. GI-2. Plant Cell Tissue Organ Cult..

[30] Budzianowska A., Kikowska M., Budzianowski J. (2024). Phenylethanoid Glycosides Accumulation and Antiradical Activity of Fractionated Extracts of *Plantago ovata* Forssk. Callus Cultures Lines. Plant Cell Tissue Organ Cult..

[31] Tomilova S.V., Kochkin D.V., Tyurina T.M., Glagoleva E.S., Labunskaya E.A., Galishev B.A., Nosov A.M. (2022). Specificity of Growth and Synthesis of Secondary Metabolites in Cultures in Vitro *Digitalis lanata* Ehrh. Russ. J. Plant Physiol..

[32] Rahamooz-Haghighi S., Bagheri K., Danafar H., Sharafi A. (2020). Tissue Culture, In Vitro Organogenesis and Regeneration of *Plantago lanceolata*. J. Apple Biotechnol. Rep..

[33] Bajaj Y.P.S., Bajaj Y.P.S. (1990). Somaclonal Variation—Origin, Induction, Cryopreservation, and Implications in Plant Breeding. Somaclonal Variation in Crop Improvement I.

[34] Kour G., Kour B., Kaul S., Dhar M.K. (2009). Genetic and Epigenetic Instability of Amplification-Prone Sequences of a Novel B Chromosome Induced by Tissue Culture in *Plantago lagopus* L.. Plant Cell Rep..

[35] De Morais Oliveira J.P., Silva A.J.D., Catrinck M.N., Clarindo W.R. (2023). Embryonic Abnormalities and Genotoxicity Induced by 2,4-Dichlorophenoxyacetic Acid during Indirect Somatic Embryogenesis in *Coffea*. Sci. Rep..

[36] Castro M., Castro S., Loureiro J. (2012). Genome Size Variation and Incidence of Polyploidy in Scrophulariaceae Sensu Lato from the Iberian Peninsula. AoB PLANTS.

[37] Suda J., Kyncl T., Jarolímová V. (2005). Genome Size Variation in Macaronesian Angiosperms: Forty Percent of the Canarian Endemic Flora Completed. Plant Syst. Evol..

[38] Wang L.-B., Dong N.-W., Wu Z.-H., Wu L.-J. (2012). Two New Compounds with Cytotoxic Activity on the Human Melanoma A375-S2 Cells from *Daphne giraldii* Callus Cells. J. Asian Nat. Prod. Res..

[39] Gjörloff Wingren A., Ziyad Faik R., Holefors A., Filecovic E., Gustafsson A. (2023). In Vitro Effects of Undifferentiated Callus Extracts from *Plantago major* L., *Rhodiola rosea* L. and *Silybum marianum* L. in Normal and Malignant Human Skin Cells. Heliyon.

[40] Jiang G., Li R.-H., Sun C., Liu Y.-Q., Zheng J.-N. (2014). Dacarbazine Combined Targeted Therapy versus Dacarbazine Alone in Patients with Malignant Melanoma: A Meta-Analysis. PLoS ONE.

[41] Koprowska K., Czyż M. (2011). Dacarbazine, a Chemotherapeutic against Metastatic Melanoma and a Reference Drug for New Treatment Modalities. Postepy Hig. Med. Dosw..

[42] Lev D.C., Ruiz M., Mills L., McGary E.C., Price J.E., Bar-Eli M. (2003). Dacarbazine Causes Transcriptional Up-Regulation of Interleukin 8 and Vascular Endothelial Growth Factor in Melanoma Cells: A Possible Escape Mechanism from Chemotherapy. Mol. Cancer Ther..

[43] VanderWalde A., Bellasea S.L., Kendra K.L., Khushalani N.I., Campbell K.M., Scumpia P.O., Kuklinski L.F., Collichio F., Sosman J.A., Ikeguchi A. (2023). Ipilimumab with or without Nivolumab in PD-1 or PD-L1 Blockade Refractory Metastatic Melanoma: A Randomized Phase 2 Trial. Nat. Med..

[44] Jung T., Haist M., Kuske M., Grabbe S., Bros M. (2021). Immunomodulatory Properties of BRAF and MEK Inhibitors Used for Melanoma Therapy—Paradoxical ERK Activation and Beyond. Int. J. Mol. Sci..

[45] Erenler R., Demirtas I., Karan T., Altun M., Gul F. (2017). Inhibitory Effect of 6,7-Dimethoxy-5-Hydroxyflavone on Human Cervix Carcinoma in Vitro. Int. J. Second. Metab..

[46] Farid M.M., Marzouk M.M., El-Shabrawy M., Salem M.A., Mounier M.M., Hussein S.R. (2019). Isoscutellarein 8, 4′-Dimethyl Ether Glycosides as Cytotoxic Agents and Chemotaxonomic Markers in *Kickxia aegyptiaca*. Biocatal. Agric. Biotechnol..

[47] Hermosaningtyas A.A., Totoń E., Lisiak N., Kruszka D., Budzianowska A., Kikowska M. (2024). Evaluation of Cytotoxic Activity of Cell Biomass from *Eryngium planum* and *Lychnis flos-cuculi* on Melanoma Cancer Cell. Molecules.

[48] Hei B., Wang J., Wu G., Ouyang J., Liu R. (2019). Verbascoside Suppresses the Migration and Invasion of Human Glioblastoma Cells via Targeting C-Met-Mediated Epithelial-Mesenchymal Transition. Biochem. Biophys. Res. Commun..

[49] Daneshforouz A., Nazemi S., Gholami O., Kafami M., Amin B. (2021). The Cytotoxicity and Apoptotic Effects of Verbascoside on Breast Cancer 4T1 Cell Line. BMC Pharmacol. Toxicol..

[50] Wu C., Chen C., Hsieh P., Lee Y., Kuo W.W., Wu R.C., Hung C., Yang Y., Lin V.C. (2021). Verbascoside Inhibits the Epithelial-mesenchymal Transition of Prostate Cancer Cells through High-mobility Group Box 1/Receptor for Advanced Glycation End-products/ TGF-β Pathway. Environ. Toxicol..

[51] Budzianowska A., Totoń E., Romaniuk-Drapała A., Kikowska M., Budzianowski J. (2023). Cytotoxic Effect of Phenylethanoid Glycosides Isolated from *Plantago lanceolata* L. *Life*
**2023**, *13*, 556. Life.

[52] Cheimonidi C., Samara P., Polychronopoulos P., Tsakiri E.N., Nikou T., Myrianthopoulos V., Sakellaropoulos T., Zoumpourlis V., Mikros E., Papassideri I. (2018). Selective Cytotoxicity of the Herbal Substance Acteoside against Tumor Cells and Its Mechanistic Insights. Redox Biol..

[53] Wu Y., Zeng M., Xu R., Zhang B., Wang S., Li B., Kan Y., Cao B., Zheng X., Feng W. (2021). Inhibitory Activity of Acteoside in Melanoma via Regulation of the ERβ-Ras/Raf1-STAT3 Pathway. Arch. Biochem. Biophys..

[54] Smith D.E., Salerno J.W. (1992). Selective Growth Inhibition of a Human Malignant Melanoma Cell Line by Sesame Oil in Vitro. Prostaglandins Leukot. Essent. Fat. Acids.

[55] De Sousa Andrade L.N., Lima T.M.D., Curi R., Castrucci A.M.D.L. (2005). Toxicity of Fatty Acids on Murine and Human Melanoma Cell Lines. Toxicol. In Vitro.

[56] Ando H., Funasaka Y., Oka M., Ohashi A., Furumura M., Matsunaga J., Matsunaga N., Hearing V.J., Ichihashi M. (1999). Possible Involvement of Proteolytic Degradation of Tyrosinase in the Regulatory Effect of Fatty Acids on Melanogenesis. J. Lipid Res..

[57] Ando H., Watabe H., Valencia J.C., Yasumoto K., Furumura M., Funasaka Y., Oka M., Ichihashi M., Hearing V.J. (2004). Fatty Acids Regulate Pigmentation via Proteasomal Degradation of Tyrosinase: A New Aspect of Ubiquitin-Proteasome Function. J. Biol. Chem..

[58] Ando H., Wen Z.-M., Kim H.-Y., Valencia J.C., Costin G.-E., Watabe H., Yasumoto K., Niki Y., Kondoh H., Ichihashi M. (2006). Intracellular Composition of Fatty Acid Affects the Processing and Function of Tyrosinase through the Ubiquitin–Proteasome Pathway. Biochem. J..

[59] Chaikul P., Lourith N., Kanlayavattanakul M. (2017). Antimelanogenesis and Cellular Antioxidant Activities of Rubber (*Hevea brasiliensis*) Seed Oil for Cosmetics. Ind. Crops Prod..

[60] Yamada H., Hakozaki M., Uemura A., Yamashita T. (2019). Effect of Fatty Acids on Melanogenesis and Tumor Cell Growth in Melanoma Cells. J. Lipid Res..

[61] Kanlayavattanakul M., Lourith N., Chaikul P. (2021). Valorization of Spent Coffee Grounds as the Specialty Material for Dullness and Aging of Skin Treatments. Chem. Biol. Technol. Agric..

