# Supplementary material for: Biotechnology Production of Cell Biomass from the Endangered Kickxia elatine (L.) Dumort: Its Untargeted Metabolomic Analysis and Cytotoxic Potential Against Melanoma Cells
Source: Biomedicines. 2025 Jun 4;13(6):1382. doi: 10.3390/biomedicines13061382 (PMC12190243; doi:10.3390/biomedicines13061382)
Supplement: Supplementary file 1 [file biomedicines-13-01382-s001.zip › biomedicines-3566448-supplementary.pdf]

Table S1 Identified metabolites in callus and cell suspension culture of *Kickxia elatine* (L.) Dumort using MS-DIAL

No	RT (min)	Formula	Adduct type	Measured m/z	Reference m/z	Metabolite name	Ontology
1	2.10	$C_4H_8O_5$	[M-H]-	135.0284	135.0299	Threonic acid	Sugar acids and derivatives
2	2.10	$C_5H_9NO_4$	[M-H]-	146.0445	146.0459	Glutamic acid	Glutamic acid and derivatives
3	2.10	$C_6H_{12}O_7$	[M-H]-	195.0501	195.051	Gluconic acid	Medium-chain hydroxy acids and derivatives
4	2.12	$C_4H_8N_4O_4$	[M-H]-	175.0461	175.0473	Allantoic acid	N-carbamoyl-alpha amino acids
5	2.15	$C_4H_6O_5$	[M-H]-	133.0127	133.0143	D-(+)-Malic acid	Beta hydroxy acids and derivatives
6	2.25	$C_6H_8O_7$	[M-H]-	191.0187	191.0197	Citrate	Tricarboxylic acids and derivatives
7	2.59	$C_{17}H_{27}N_3O_{17}P_2$	[M-H]-	606.0746	606.0743	Uridine 5'-diphospho-N-acetylglucosamine	Pyrimidine nucleotide sugars
8	2.59	$C_{15}H_{24}N_2O_{17}P_2$	[M-H]-	565.048	565.0477	Uridine 5'-diphosphogalactose	Pyrimidine nucleotide sugars
9	2.60	$C_{10}H_{13}N_5O_4$	[M-H]-	266.0895	266.0895	Deoxyguanosine	Purine 2'-deoxyribonucleosides
10	2.60	$C_4H_8O_5$	[M-H]-	135.0284	135.0299	Threonic acid	Sugar acids and derivatives
11	2.60	$C_6H_{10}O_5$	[M-H]-	161.0442	161.0456	3-hydroxy-3-methylglutarate	Hydroxy fatty acids
12	2.61	$C_9H_{13}N_2O_9P$	[M-H]-	323.0288	323.0286	Uridine monophosphate	Pyrimidine ribonucleoside monophosphates
13	2.61	$C_6H_{10}O_4$	[M-H]-	145.0604	145.0506	Adipic acid	Medium-chain fatty acids
14	2.61	$C_6H_8O_7$	[M-H]-	191.0188	191.0197	Citrate	Tricarboxylic acids and derivatives
15	2.62	$C_{10}H_{13}N_5O_5$	[M-H]-	282.0912	282.0844	Guanosine	Purine nucleosides
16	2.62	$C_6H_{12}O_7$	[M-H]-	195.05	195.051	Gluconic acid	Medium-chain hydroxy acids and derivatives
17	2.62	$C_{12}H_{22}O_{11}$	[M-H]-	341.1087	341.109	Trehalose	O-glycosyl compounds
18	2.64	$C_4H_6O_5$	[M-H]-	133.0128	133.0143	(S)-Malate	Beta hydroxy acids and derivatives
19	2.64	$C_{10}H_{12}N_4O_6$	[M-H]-	283.0683	283.0684	Xanthoside	Purine nucleosides
20	2.71	$C_8H_{16}N_2O_3$	[M-H]-	187.1079	187.1088	Gly-Leu	Peptides
21	2.71	$C_9H_{18}N_2O_3$	[M-H]-	201.1237	201.1245	Ala-Ile	Dipeptides
22	2.78	$C_9H_{11}NO_2$	[M-H]-	164.0704	164.0717	Phenylalanine	Phenylalanine and derivatives
23	2.79	$C_5H_8O_4$	[M-H]-	131.0336	131.035	Glutarate	Dicarboxylic acids and derivatives
24	2.83	$C_{11}H_{15}N_5O_5$	[M-H]-	296.1	296.1	2'-O-Methylguanosine	Purine nucleosides
25	2.83	$C_8H_8O_4$	[M-H]-	167.0451	167.035	Homogenistic acid	2(hydroxyphenyl)acetic acids
26	2.84	$C_{10}H_9NO_3$	[M-H]-	190.0501	190.051	(2-oxo-2,3-dihydro-1H-indol-3-yl)acetic acid	Indolyl carboxylic acids and derivatives

27	2.87	$C_6H_8O_7$	[M-H]-	191.0188	191.0197	Citrate	Tricarboxylic acids and derivatives
28	2.87	$C_4H_6O_5$	[M-H]-	133.0128	133.0143	D-(+)-Malic acid	Beta hydroxy acids and derivatives
29	3.06	$C_6H_{10}O_5$	[M-H]-	161.0443	161.0456	Hydroxymethylglutaric acid	Hydroxy fatty acids
30	3.19	$C_9H_{10}N_2O_5$	[M-H]-	225.0511	225.0517	3-nitro-L-tyrosine	Tyrosine and derivatives
31	3.22	$C_9H_{17}NO_5$	[M-H]-	218.1029	218.1034	Pantothenate	Secondary alcohols
32	3.29	$C_{14}H_{18}N_2O_6$	[M-H]-	309.1096	309.1092	gamma-Glutamyltyrosine	Tyrosine and derivatives
33	3.36	$C_{12}H_{17}N_5O_5$	[M-H]-	310.1159	310.1157	N2,N2-Dimethylguanosine	Purine nucleosides
34	3.59	$C_6H_5NO_4$	[M-H]-	154.0133	154.0146	Citrazinc acid	Pyridinecarboxylic acids
35	3.61	$C_8H_8O_4$	[M-H]-	167.0338	167.035	2,4,6-Trihydroxyacetophenone	Alkyl-phenylketones
36	3.81	$C_9H_{17}NO_5$	[M-H]-	218.1025	218.1034	Pantothenate	Secondary alcohols
37	3.81	$C_{13}H_{16}O_9$	[M-H]-	315.0725	315.0721	Benzoic acid + 2O, *O*-Hex	Phenolic glycosides
38	3.85	$C_5H_5N_5$	[M-H]-	134.0457	134.0472	Adenine	6-aminopurines
39	3.85	$C_4H_6O_5$	[M-H]-	133.0128	133.0143	D-(+)-Malic acid	Beta hydroxy acids and derivatives
40	3.86	$C_{11}H_{15}N_5O_3S$	[M-H]-	296.0824	296.0823	5'-Deoxy-5'-Methylthioadenosine	5'-deoxy-5'-thionucleosides
41	3.87	$C_9H_{11}NO_2$	[M-H]-	164.0706	164.0717	Phenylalanine	Phenylalanine and derivatives
42	3.87	$C_6H_8O_7$	[M-H]-	191.0187	191.0197	Citric acid	Tricarboxylic acids and derivatives
43	3.89	$C_{11}H_{12}N_2O_2$	[M-H]-	203.0818	203.0826	Tryptophan	Indolyl carboxylic acids and derivatives
44	3.90	$C_6H_{11}NO_3S$	[M-H]-	176.0376	176.0387	N-Formylmethionine	Methionine and derivatives
45	4.03	$C_{10}H_{13}NO_4$	[M-H]-	210.0765	210.0772	Methoxytyrosine	Tyrosine and derivatives
46	4.07	$C_8H_{14}O_5$	[M-H]-	189.0759	189.0768	Hydroxysuberic acid	Medium-chain hydroxy acids and derivatives
47	4.09	$C_{11}H_{20}N_2O_5$	[M-H]-	259.1187	259.1299	gamma-Glutamylleucine	Dipeptides
48	4.27	$C_7H_{13}NO_3S$	[M-H]-	190.0534	190.0543	N-acetyl-DL-methionine	Methionine and derivatives
49	4.28	$C_7H_6O_4$	[M-H]-	153.0179	153.0193	3,4-Dihydroxybenzoic acid	Hydroxybenzoic acid derivatives
50	4.34	$C_{14}H_{20}O_7$	[M-H]-	299.1137	299.1136	Salidroside	O-glycosyl compounds
51	4.36	$C_9H_{10}O4$	[M-H]-	181.0496	181.0506	4-Hydroxyphenyllactic acid	Phenylpropanoic acids
52	4.41	$C_8H_8O_3$	[M-H]-	151.0389	151.0401	P-anisic acid	P-methoxybenzoic acids and derivatives
53	4.50	$C_7H_{12}O_5$	[M-H]-	175.06	175.0612	2-Isopropylmalic acid	Hydroxy fatty acids
54	4.64	$C_{19}H_{28}O_{11}$	[M-H]-	431.1556	431.1559	(2R,3S,4S,5R,6R)-5-[(2S,3R,4R)-3,4-dihydroxy-4-(hydroxymethyl)oxolan-2-yl]oxy-2-(hydroxymethyl)-6-[2-	Phenylpropanoid

						(4-hydroxyphenyl)ethoxy]oxane-3,4-diol	
55	4.66	$C_8H_8O_3$	[M-H]-	151.0387	151.0401	P-anisic acid	P-methoxybenzoic acids and derivatives
56	4.69	$C_9H_{11}NO_3$	[M-H]-	180.0655	180.0666	Tyrosine	Tyrosine and derivatives
57	4.69	$C_{12}H_{12}N_2O_2$	[M-H]-	215.0819	215.0826	L-1,2,3,4-Tetrahydro-beta-carboline-3-carboxylic acid	Beta carbolines
58	5.02	$C_6H_{12}O_3$	[M-H]-	131.0699	131.0714	Hydroxyisocaproic acid	Hydroxy fatty acids
59	5.03	$C_{14}H_{18}N_2O_5$	[M-H]-	293.1242	293.1143	Glutamylphenylalanine	Phenylalanine and derivatives
60	5.04	$C_7H_{12}O_4$	[M-H]-	159.065	159.0663	Pimelic acid	Medium-chain fatty acids
61	5.07	$C_{15}H_{18}O_9$	[M-H]-	341.0877	341.0878	Caffeic acid hexoside	Hydroxycinnamic acid glycosides
62	5.11	$C_6H_{10}O_3$	[M-H]-	129.0542	129.0557	Ketoleucine	Short-chain keto acids and derivatives
63	5.13	$C_{15}H_{20}O_8$	[M-H]-	327.1087	327.1086	1-(4-hydroxyphenyl)-3-[(2R,3R,4S,5S,6R)-3,4,5-trihydroxy-6-(hydroxymethyl)oxan-2-yl]oxypropan-1-one	Fatty acyl glycosides of mono- and disaccharides
64	5.20	$C_{12}H_{12}N_2O_2$	[M-H]-	215.0819	215.0826	(3S)-1H,2H,3H,4H,9H-pyrido(3,4-b)indole-3-carboxylic acid	Beta carbolines
65	5.21	$C_{10}H_{13}NO_4$	[M-H]-	210.0764	210.0772	Methoxytyrosine	Tyrosine and derivatives
66	5.32	$C_9H_8O_4$	[M-H]-	179.034	179.035	Caffeic acid	Hydroxycinnamic acids
67	5.38	$C_6H_{12}O_3$	[M-H]-	131.0699	131.0714	Hydroxyisocaproic acid	Hydroxy fatty acids
68	5.40	$C_{17}H_{20}N_4O_6$	[M-H]-	375.131	375.131	Riboflavin	Flavins
69	5.46	$C_{27}H_{33}N_9O_{15}P_2$	[M-H]-	784.1503	784.1498	Flavin adenine dinucleotide	Flavin nucleotides
70	5.50	$C_8H_{15}NO_3$	[M-H]-	172.0969	172.0979	N-acetyl-L-leucine	Leucine and derivatives
71	5.54	$C_4H_7NO_4$	[M-H]-	132.0288	132.0302	Aspartate	Aspartic acid and derivatives
72	5.57	$C_9H_{10}O_3$	[M-H]-	165.0544	165.0557	3-Phenyllactic acid	Phenylpropanoic acids
73	5.62	$C_6H_{12}O_3$	[M-H]-	131.0698	131.0714	Hydroxyisocaproic acid	Hydroxy fatty acids
74	5.71	$C_8H_{15}NO_3$	[M-H]-	172.0967	172.0979	N-Acetylleucine	Leucine and derivatives
75	5.72	$C_9H_{11}NO_2$	[M-H]-	164.0705	164.0717	Phenylalanine	Phenylalanine and derivatives
76	5.80	$C_9H_8O_4$	[M-H]-	179.0339	179.035	Caffeic acid	Hydroxycinnamic acids
77	5.81	$C_{19}H_{18}O_{11}$	[M-H]-	421.078	421.0776	Mangiferin	Xanthones
78	5.83	$C_8H_{14}O_5$	[M-H]-	189.0758	189.0768	Hydroxysuberic acid	Medium-chain hydroxy acids and derivatives

79	5.84	$C_{19}H_{28}O_{10}$	[M-H]-	415.1614	415.161	(2R,3S,4S,5R,6R)-2-[[(2S,3R,4R)-3,4-dihydroxy-4-(hydroxymethyl)oxolan-2-yl]oxymethyl]-6-(2-phenylethoxy)oxane-3,4,5-triol	Phenylpropanoid
80	5.90	$C_{11}H_{20}O_4$	[M-H]-	215.1282	215.1289	Undecanedioic acid	Medium-chain fatty acids
81	6.00	$C_{11}H_{13}N_3O$	[M-H]-	202.1076	202.0986	Tryptophanamide	Tryptamines and derivatives
82	6.07	$C_{11}H_{13}NO_3$	[M-H]-	206.0813	206.0823	N-acetyl-L-phenylalanine	Phenylalanine and derivatives
83	6.08	$C_9H_{10}O_3$	[M-H]-	165.0545	165.0557	3-Phenyllactic acid	Phenylpropanoic acids
84	6.10	$C_9H_{11}NO_2$	[M-H]-	164.0705	164.0717	Phenylalanine	Phenylalanine and derivatives
85	6.11	$C_9H_{10}O_4$	[M-H]-	181.0495	181.0506	b-Orcinolcarboxylic acid	Hydroxybenzoic acid derivatives
86	6.15	$C_9H_{16}O_4$	[M-H]-	187.0966	187.0976	Azelaic acid	Medium-chain fatty acids
87	6.18	$C_{10}H_{18}O_5$	[M-H]-	217.1074	217.1082	3-Hydroxysebacic acid	Medium-chain hydroxy acids and derivatives
88	6.19	$C_{35}H_{46}O_{20}$	[M-H]-	785.2514	785.251	Echinacoside	Phenylpropanoid
89	6.26	$C_{11}H_{13}NO_3$	[M-H]-	206.0814	206.0823	N-acetyl-L-phenylalanine	Phenylalanine and derivatives
90	6.31	$C_9H_{11}NO_2$	[M-H]-	164.0704	164.0717	Phenylalanine	Phenylalanine and derivatives
91	6.31	$C_{34}H_{44}O_{19}$	[M-H]-	755.2402	755.2404	Lavandulifolioside	Phenylpropanoid
92	6.60	$C_5H_5N_5$	[M-H]-	134.0457	134.0472	Adenine	6-aminopurines
93	6.63	$C_{10}H_7NO_3$	[M-H]-	188.0342	188.0353	Kynurenic acid	Quinoline carboxylic acids
94	6.67	$C_{29}H_{36}O_{15}$	[M-H]-	623.1978	623.1981	Acteoside	Phenylpropanoid
95	6.69	$C_{12}H_{22}O_6$	[M-H]-	261.1343	261.1344	9-(2,3-dihydroxypropoxy)-9-oxononanoic acid	Medium-chain fatty acids
96	6.71	$C_{10}H_{18}O_5$	[M-H]-	217.1074	217.1082	Hydroxysebacic acid	Medium-chain hydroxy acids and derivatives
97	6.76	$C_{35}H_{46}O_{19}$	[M-H]-	769.2556	769.256	[6-[2-(3,4-dihydroxyphenyl)ethoxy]-2-(hydroxymethyl)-4-(3,4,5-trihydroxy-6-methyloxan-2-yl)oxy-5-(3,4,5-trihydroxyoxan-2-yl)oxyoxan-3-yl] (E)-3-(4-hydroxy-3-methoxyphenyl)prop-2-enoate	Phenylpropanoid
98	6.92	$C_9H_8O_3$	[M-H]-	163.0389	163.0401	trans-4-Coumaric acid	Hydroxycinnamic acids
99	6.92	$C_{12}H_{22}O_6$	[M-H]-	261.1342	261.1344	9-(2,3-dihydroxypropoxy)-9-oxononanoic acid	Medium-chain fatty acids
100	6.93	$C_9H_{16}O_4$	[M-H]-	187.0965	187.0976	Azelaic acid	Medium-chain fatty acids
101	6.99	$C_{17}H_{18}O_9$	[M-H]-	365.0877	365.0878	1-hydroxy-4-[(2S,3R,4S,5S,6R)-3,4,5-trihydroxy-6-	Phenolic glycosides

						(hydroxymethyl)oxan-2-yl]oxynaphthalene-2-carboxylic acid	
102	7.09	$C_{29}H_{36}O_{15}$	[M-H]-	623.1979	623.1981	Isoacteoside	Phenylpropanoid
103	7.19	$C_{30}H_{37}O_{15}$	[M-H]-	637.2136	637.2127	((2R,3R,4R,5R,6R)-6-(2-(3,4-dihydroxyphenyl)ethoxy)-5-hydroxy-2-(hydroxymethyl)-4-((2S,3R,4R,5R,6S)-3,4,5-trihydroxy-6-methyloxan-2-yl)oxyoxan-3-yl) (E)-3-(4-hydroxy-3-methoxyphenyl)prop-2-enoate	Phenylpropanoid
103	7.30	$C_9H_7NO_2$	[M-H]-	160.0391	160.0404	Indole-3-carboxylic acid	Indolecarboxylic acids and derivatives
104	7.35	$C_{10}H_{18}O_4$	[M-H]-	201.1123	201.1132	Decanedioic acid	Medium-chain fatty acids
105	7.39	$C_9H_{16}O_4$	[M-H]-	187.0965	187.0976	Azelaic acid	Medium-chain fatty acids
106	7.78	$C_9H_{16}O_4$	[M-H]-	187.0965	187.0976	Azelaic acid	Medium-chain fatty acids
107	8.08	$C_{10}H_{18}O_4$	[M-H]-	201.1122	201.1132	Decanedioic acid	Medium-chain fatty acids
108	8.13	$C_{16}H_{32}O_5$	[M-H]-	303.218	303.2177	Aleuretic Acid	Long-chain fatty acids
109	8.47	$C_{10}H_{18}O_4$	[M-H]-	201.1123	201.1132	Decanedioic acid	Medium-chain fatty acids
110	8.48	$C_9H_{16}O_3$	[M-H]-	171.1014	171.1027	FA 9:1+1O	Medium-chain fatty acids
111	8.70	$C_{16}H_{32}O_5$	[M-H]-	303.2178	303.2177	Aleuretic Acid	Long-chain fatty acids
112	8.71	$C_9H_{16}O_4$	[M-H]-	187.0966	187.0976	Azelaic acid	Medium-chain fatty acids
113	9.11	$C_9H_{10}O_3$	[M-H]-	165.0545	165.0557	Methyl 2-methoxybenzoate	O-methoxybenzoic acids and derivatives
114	9.17	$C_{18}H_{34}O_5$	[M-H]-	329.2335	329.2333	(Z)-5,8,11-trihydroxyoctadec-9-enoic acid	Long-chain fatty acids
115	9.47	$C_{11}H_{20}O_4$	[M-H]-	215.1281	215.1289	Undecanedioic acid	Medium-chain fatty acids
116	9.81	$C_{12}H_{22}O_4$	[M-H]-	229.1441	229.1445	Dodecanedioic acid	Medium-chain fatty acids
117	9.82	$C_{18}H_{34}O_5$	[M-H]-	329.2333	329.2333	(Z)-5,8,11-trihydroxyoctadec-9-enoic acid	Long-chain fatty acids
118	10.41	$C_{12}H_{22}O_4$	[M-H]-	229.1439	229.1445	Dodecanedioic acid	Medium-chain fatty acids
119	10.61	$C_{18}H_{30}O_4$	[M-H]-	309.2074	309.2071	FA 18:3+2O	Lineolic acids and derivatives
120	10.70	$C_{18}H_{34}O_5$	[M-H]-	329.2336	329.2333	FA 18:1+3O	Long-chain fatty acids
121	11.04	$C_{12}H_{22}O_4$	[M-H]-	229.1439	229.1445	Dodecanedioic acid	Medium-chain fatty acids
122	11.30	$C_{13}H_{24}O_4$	[M-H]-	243.1597	243.1602	Tridecanedioic acid	Long-chain fatty acids
123	11.33	$C_{18}H_{34}O_5$	[M-H]-	329.2333	329.2333	(Z)-5,8,11-trihydroxyoctadec-9-enoic acid	Long-chain fatty acids

124	11.42	$C_{15}H_{22}O_2$	[M+CH3COO]-	293.1761	293.1758	2-[(2R,8S,8aR)-8,8a-dimethyl-2,3,5,6,7,8-hexahydro-1H-naphthalen-2-yl]prop-2-enoic acid	Eremophilane, 8,9-secoeremophilane and furoeremophilane sesquiterpenoids
125	11.62	$C_{18}H_{34}O_4$	[M-H]-	313.2385	313.2384	Octadecanedioic acid	Long-chain fatty acids
126	11.71	$C_{18}H_{30}O_4$	[M-H]-	309.2072	309.2071	(9S,10E,12Z,15Z)-9-hydroperoxyoctadeca-10,12,15-trienoic acid	Lineolic acids and derivatives
127	11.90	$C_{18}H_{34}O_5$	[M-H]-	329.2336	329.2333	(Z)-5,8,11-trihydroxyoctadec-9-enoic acid	Long-chain fatty acids
128	12.03	$C_{18}H_{34}O_5$	[M-H]-	329.2335	329.2333	(Z)-9,12,13-trihydroxyoctadec-15-enoic acid	Long-chain fatty acids
129	12.45	$C_9H_{16}O_4$	[M-H]-	187.0966	187.0976	Azelaic acid	Medium-chain fatty acids
130	12.64	$C_{18}H_{34}O_5$	[M-H]-	329.2335	329.2333	(Z)-5,8,11-trihydroxyoctadec-9-enoic acid	Long-chain fatty acids
131	12.73	$C_9H_{16}O_4$	[M-H]-	187.0965	187.0976	Azelaic acid	Medium-chain fatty acids
132	12.88	$C_{15}H_{22}O_3$	[M-H]-	249.1492	249.1496	2-[(2S,4aR,8aS)-2-hydroxy-4a-methyl-8-methylidene-3,4,5,6,7,8a-hexahydro-1Hnaphthalen-2-yl]prop-2-enoic acid	Eudesmane, isoeudesmane or cycloeudesmane sesquiterpenoids
133	13.03	$C_{18}H_{30}O_4$	[M-H]-	309.2073	309.2071	FA 18:3+2O	Long-chain fatty acids
134	13.16	$C_{18}H_{32}O_2$	[M-H]-	279.2327	279.2329	Linoleic acid	Lineolic acids and derivatives
135	13.24	$C_{18}H_{32}O_3$	[M-H]-	295.2277	295.2279	9-HODE	Lineolic acids and derivatives
136	13.36	$C_{18}H_{30}O_3$	[M-H]-	293.2125	293.2122	9-HOTrE	Lineolic acids and derivatives
137	13.40	$C_{18}H_{30}O_4$	[M-H]-	309.2074	309.2071	FA 18:3+2O	Long-chain fatty acids
138	13.46	$C_{27}H_{46}O_9$	[M+HCOO]-	559.3121	559.3124	Panaxcerol B	Glycoglycerolipid
139	13.52	$C_{16}H_{22}O_4$	[M-H]-	277.1443	277.1445	Di-n-butyl phthalate	Benzoic acid esters
140	13.53	$C_{18}H_{34}O_5$	[M-H]-	329.2336	329.2333	FA 18:1+3O	Long-chain fatty acids
141	13.53	$C_{18}H_{32}O_3$	[M-H]-	295.2278	295.2279	9-HODE	Lineolic acids and derivatives
142	14.09	$C_{14}H_{28}O_3$	[M-H]-	243.196	243.1966	beta-Hydroxymyristic acid	Long-chain fatty acids
143	14.10	$C_{18}H_{32}O_3$	[M-H]-	295.2277	295.2279	9-HODE	Lineolic acids and derivatives
144	14.77	$C_{18}H_{34}O_3$	[M-H]-	297.2434	297.2435	FA 18:1+1O	Lineolic acids and derivatives
145	14.90	$C_5H_{12}O_5$	[M-H]-	151.0593	151.0612	Xylitol	Sugar alcohols
146	14.91	$C_{18}H_{32}O_2$	[M-H]-	279.2327	279.2329	Linoleic acid	Lineolic acids and derivatives
147	15.10	$C_{18}H_{32}O_3$	[M-H]-	295.2278	295.2279	9-HODE	Lineolic acids and derivatives
148	15.32	$C_{15}H_{22}O_3$	[M-H]-	249.1526	249.1496	2-[(2S,4aR,8aS)-2-hydroxy-4a-methyl-8-methylidene-3,4,5,6,7,8a-hexahydro-1H-	Eudesmane, isoeudesmane or cycloeudesmane sesquiterpenoids

						naphthalen-2-yl]prop-2-enoic acid	
149	15.64	$C_{18}H_{32}O_3$	[M-H]-	295.2278	295.2279	9-HODE	Lineolic acids and derivatives
150	15.67	$C_{15}H_{22}O_3$	[M-H]-	249.1492	249.1496	2-[(1S,2S,4aR,8aS)-1-hydroxy-4a-methyl-8-methylidene-1,2,3,4,5,6,7,8a-octahydronaphthalen-2-yl]prop-2-enoic acid	Eudesmane, isoeudesmane or cycloeudesmane sesquiterpenoids
